# Preoperative left ventricular ejection fraction and left atrium reverse remodeling after mitral regurgitation surgery

**DOI:** 10.1186/1476-7120-12-45

**Published:** 2014-11-06

**Authors:** Lucia R Machado, Zilda M Meneghelo, David CS Le Bihan, Rodrigo BM Barretto, Antonio C Carvalho, Valdir A Moises

**Affiliations:** Instituto Dante Pazzanese de Cardiologia, São Paulo, SP Brazil; Escola Paulista de Medicina, Universidade Federal de São Paulo, São Paulo, SP Brazil; Disciplina de Cardiologia, Rua Napoleão de Barros, 715, São Paulo, SP CEP: 04024-002 Brazil

**Keywords:** Mitral valve insufficiency, Mitral valve surgery, Left atrial remodeling, Left atrial volume

## Abstract

**Background:**

Left atrium enlargement has been associated with cardiac events in patients with mitral regurgitation (MR). Left atrium reverse remodeling (LARR) occur after surgical correction of MR, but the preoperative predictors of this phenomenon are not well known. It is therefore important to identify preoperative predictors for postoperative LARR.

**Methods:**

We enrolled 62 patients with chronic severe MR (prolapse or flail leaflet) who underwent successful mitral valve surgery (repair or replacement); all with pre- and postoperative echocardiography. LARR was defined as a reduction in left atrium volume index (LAVI) of ≥25%. Stepwise multiple regression analysis was used to identify independent predictors of LARR.

**Results:**

LARR occurred in 46 patients (74.2%), with the mean LAVI decreasing from 85.5 mL/m^2^ to 49.7 mL/m^2^ (*p* <0.001). These patients had a smaller preoperative left ventricular systolic volume (*p* =0.022) and a higher left ventricular ejection fraction (LVEF) (*p* =0.034). LVEF was identified as the only preoperative variable significantly associated with LARR (odds ratio, 1.086; 95% confidence interval, 1.002–1.178). A LVEF cutoff value of 63.5% identified patients with LARR of ≥25% with a sensitivity of 71.7% and a specificity of 56.3%.

**Conclusions:**

LARR occurs frequently after mitral valve surgery and is associated with preoperative LVEF higher than 63.5%.

## Introduction

Both left atrial enlargement and remodeling are compensatory mechanisms in patients with chronic severe mitral regurgitation (MR). These changes allow the left atrium to accommodate the regurgitant volume without increased pressure [1–3]. It is recognized that left atrial enlargement is a marker of major cardiovascular events such as atrial fibrillation, stroke, and death in patients with cardiac disease [4–8]. In patients with chronic severe MR, left atrial size seems to be an important predictor of outcome, either during conservative treatment or after mitral valve surgery [3, 9–11]. A decrease in left atrial size, or reverse remodeling, has been observed after mitral valve surgery [12–17]. These prior studies included patients with different causes of MR, analyzed left atrial size using different techniques, and found different pre- and postoperative factors related to postoperative left atrial reverse remodeling (LARR). Different definitions of LARR after MR surgery have also been used; some considered any reduction value and others no more than 15% [12–17]. Low values may be very sensitive and may not have great hemodynamic meaning. We hypothesized that larger postoperative LARR would represent a stronger hemodynamic impact by surgery and have different predictor factors. The objectives of our study were to assess the changes in left atrial size after surgery for MR and to verify the preoperative parameters that may be associated with or predictive of a reduction in left atrial volume index (LAVI) of at least 25%.

## Methods

### Patients

We prospectively enrolled 62 patients with degenerative (prolapse or flail leaflet) chronic severe MR who were scheduled to undergo mitral valve surgery (repair or replacement). The ethical committee review board of both participating institutions approved the study, and all patients provided informed consent (Research Ethical Committee: Escola Paulista de Medicina and Instituto Dante Pazzanese de Cardiologia). Patients were excluded if they had functional or acute MR, associated moderate or severe disease of another cardiac valve, coronary artery disease, congenital heart disease, infective endocarditis, acute rheumatic fever, stage 2 or 3 systemic hypertension, or a past history of mitral-valve surgery. Patients who had greater than mild MR after surgery were also excluded. Pre- and postoperative assessment included the patients’ clinical history, physical examination, electrocardiography, chest radiography, and echocardiography. Recommendations for coronary angiography and mitral-valve surgery (valvular repair vs. replacement) were made according to international guidelines [18, 19].

### Echocardiography

All patients had a comprehensive transthoracic echocardiogram prior to surgery (Vivid7®; GE Vingmed Ultrasound, Horton, Norway), using a 2–4 MHz multifrequential transducer. Echocardiography was repeated between 2 and 10 months after surgery. Patient position, echocardiographic imaging planes, and measures were performed as recommended by the American Society of Echocardiography [20]. Two experienced echocardiographers acquired and analyzed all images. The left ventricular diastolic and systolic diameters were measured using M-mode or 2-dimensional echocardiography. The left ventricular systolic volume, diastolic volume, and ejection fraction (LVEF) were obtained using the biplane Simpson method. The left atrial volume was estimated using the biplane area-length method and expressed in milliliters (mL). LAVI was determined by dividing left atrial volume (mL) by body surface area (m^2^). LARR was defined as a reduction of ≥25% in LAVI on postoperative echocardiography.

The morphologic and functional characteristics of the mitral valve were analyzed to determine the etiology of each patient’s disease. The severity of MR was estimated using the flow convergence method [21]. Severe MR was defined as a regurgitant volume ≥60 mL/beat and an effective regurgitant orifice area ≥0.40 cm^2^. For all measurements, diastole was defined as the beginning of the QRS complex, and systole was defined as the peak of the T wave in the same cardiac cycle. The final value of each variable was the mean of 3 measurements in different cardiac cycles.

### Statistical analysis

Continuous variables are presented as mean values ± standard deviation, and categorical variables as absolute numbers and percentages. The normal distribution of continuous variables was analyzed using the Kolmogorov-Smirnov test. The association between categorical variables was assessed using either Pearson’s chi-square or Fisher’s exact test. To compare preoperative and postoperative variables, the paired Student’s t-test or the Wilcoxon test, were used. Comparison between groups with and without LARR was done using either Student’s t-test or the Mann–Whitney test. Stepwise multiple regression analysis using the backward elimination method was used to determine preoperative variables associated with LARR. The Yolden criteria were used to identify the cutoff value of LVEF that predicted LARR ≥25% [22]. Intra- and interobserver variability was assessed by means of percentual difference between measurements, the paired t-test, and the Pearson correlation coefficient. The significance level of all tests was set at 5%.

## Results

The baseline characteristics of the patients are presented in Table 1. Thirty-four patients (54.8%) underwent mitral valve repair and 28 (45.2%) underwent valve replacement, using 19 biological and 9 mechanical prostheses. Surgical atrial reduction was not performed in any patients included in the study. In the postoperative evaluation, 52 patients were in sinus rhythm, and 10 in atrial fibrillation. Sixty (98.8%) patients had a preoperative LAVI ≥40 mL/m^2^. The LAVI decreased from a preoperative mean of 85.5 mL/m^2^ to a postoperative mean of 49.6 mL/m^2^ (*p* <0.001); the mean reduction was 39%. LARR was observed in 46 (74.2%) patients.

**Table 1:** **Baseline patient characteristics**

Variables	n (%)
Male sex	37 (59.7)
Body surface area (m^2^); mean ± SD	1.73 ± 0.19
Stage 1 systemic hypertension	37 (59.7)
Rhythm:	
Sinus	50 (80.6)
Atrial fibrillation	12 (19.4)
Functional class:	
II	43 (69.4)
III / IV	19 (30,6)
Medications:	
Diuretic	54 (84.4)
ACE inhibitor	38 (59.4)
Beta blocker	25 (39.1)
Calcium channel blocker	9 (14.1)
Digoxin	9 (14.1)
Amiodarone	1 (1.6)
Aspirin	13 (20.3)
Oral anticoagulant	3 (4.7)

ACE: angiotensin converter enzyme; SD: standard deviation.

Categorical variables such as sex, age ≥ or <50 years old, the presence or absence of systemic hypertension, functional class of I/II or III/IV, type of surgery performed (repair or replacement), postoperative mean diastolic gradient and preoperative cardiac rhythm (sinus or atrial fibrillation) were not significantly associated with LARR, with *p*-values ranging from 0.14 to 1.0. All echocardiographic variables, including LVEF, decreased significantly after surgery, but the difference in left ventricular systolic volume was not statistically significant (Table 2). Patients with LARR had smaller preoperative left ventricular systolic volume values and higher LVEF values (*p* =0.022, 0.034, respectively); other preoperative variables were not significantly different between patients with or without LARR (Table 3). Stepwise multiple regression analysis revealed that LVEF was the only preoperative variable significantly associated with LARR (odds ratio, 1.086; 95% confidence interval, 1.002–1.178). A cutoff value of 63.5% for preoperative LVEF identified patients with LARR ≥25% after surgery, with a sensitivity of 72% and a specificity of 56%.

**Table 2:** **Echocardiographic variables**

Variables	Before surgery	After surgery	***P-***value
LAVI (mL/m^2^)	85.5 ± 36.8	49.7 ± 25.1	< 0.001
LVdD (mm)	61.0 ± 5.3	52.4 ± 5.5	< 0.001
LVsD (mm)	38.3 ± 5.2	35.3 ± 6.0	< 0.001
LVdV (mL)	133.8 ± 40.1	98.2 ± 32.1	< 0.001
LVsV (mL)	46.5 ± 23.3	41.6 ± 19.8	0.23
LVEF (%)	66.1 ± 7.5	59.1 ± 9.1	< 0.001

Values given as mean ± standard deviation.

LAVI: left atrium volume index; LVdD: left ventricle diastolic diameter; LVsD: left ventricle systolic diameter; LVdV: left ventricle diastolic volume; LVsV: left ventricle systolic volume; LVEF: left ventricle ejection fraction.

**Table 3:** **Comparison of age and preoperative echocardiographic variables of patients with or without postoperative left atrium reverse remodeling**

Variable	LAVI reduction <25% (n = 16)	LAVI reduction ≥25% (n = 46)	***p***-value
Age (years)	50.3 ± 17.3	53.3 ± 14.3	0.42
LAVI (mL/m^2^)	77.3 ± 39	88.4 ± 36	0.15
LVdD (mm)	61,6 ± 5.4	60.8 ± 5.3	0.75
LVsD (mm)	39.8 ± 5.0	37.8 ± 5.1	0.25
LVdV (mL)	151 ± 46	128 ± 37	0.050
LVsV (mL)	60 ± 28	42 ± 20	0.022
LVEF (%)	63 ± 9.0	67 ± 6.7	0.034
RV (mL)	88 ± 27	96 ± 45	0.88
EROA (cm^2^)	0.56 ± 0.18	0.60 ± 0.24	0.83

Values given as mean ± standard deviation.

EROA: effective regurgitant orifice area; LAVI: left atrium volume index; LVdD: left ventricle diastolic diameter; LVsD: left ventricle systolic diameter; LVdV: left ventricle diastolic volume; LVsV: left ventricle systolic volume; LVEF: left ventricle ejection fraction; RV: mitral valve regurgitant volume per beat.

Analysis of intra- and interobserver variability showed a high and significant correlation in LAVI measures (r =0.97; *p* <0.0001) with a mean difference of 8.7% (*p* =0.10). The mean difference between the 2 LAVI measurements by the same observer was 9.3% (*p* =0.10) and the measurements were highly correlated (r =0.98; *p* <0.0001).

## Discussion

We found that LARR occurs frequently after surgery for chronic severe MR and that preoperative LVEF is the only variable significantly associated with a postoperative LAVI reduction of at least 25%.

The percentage of patients in our study with LARR after mitral valve repair or replacement is similar to other study populations previously described [12–17]. Many factors have been reported as determinants of LARR after mitral regurgitation surgery. These include MR etiology, patient age, preoperative LAVI, cardiac rhythm, mitral valve double dysfunction with predominant MR, systemic arterial pressure, diastolic mitral pressure gradient, postoperative reduction in left ventricle diastolic volume, and residual mitral regurgitation after surgery [13–17]. Prior studies differed from ours regarding the etiology of MR, the criteria used to define LARR, the techniques used to analyze left atrial size, and the statistical methods employed. In our study, a preoperative LVEF of 63.5% or higher identified those patients with at least 25% LARR. This is interesting, since LVEF in MR is an important prognostic factor for postoperative LVEF and functional class, and a determinant for surgical correction in asymptomatic patients. A LVEF of 63.5% is close to the 60% recommended as the threshold for surgery in most guidelines and similar to the finding of a recently published study [18, 19, 23, 24].

Left atrial size in patients with chronic mitral regurgitation increases with the severity of the regurgitant volume and with the duration and progression of the disease [1–3]. Also influencing left atrial dilation, LVEF is known to deteriorate in patients with advanced MR, and diastolic dysfunction may occur [2, 3]. As long as left atrial dilation persists or progresses, interstitial wall fibrosis and hypertrophy may develop and make reversal remodeling after surgery less likely [25–27] This may be a reason for the observed association of better preoperative LVEF with postoperative LARR in our study. After successful mitral-valve surgery with reduction in left ventricular volume, a decline in left ventricular filling pressure may have occurred, facilitating left atrium emptying and reverse remodeling.

As suggested by recent study, reducing mitral-regurgitation volume may be an important determinant of LARR after mitral-valve intervention [23]. This may have been an important reason for the observed LAVI reduction in the present study, since all patients had severe preoperative regurgitant volume and no more than mild postoperative mitral regurgitation. These were predefined conditions to select patients for the study and may explain why both were not associated to LARR.

Some studies define LARR as a reduction of at least 15% in LAVI, but others do not define a cutoff value [12–17]. Failing to use a criterion, or using an inadequate cutoff value, may mean that the observed reduction fell within the range of inter- and intraobserver variability, or that the reduction had a small degree of clinical significance and consequently influenced both the quantitative identification of LARR and the determination of predictor factors. Our use of a reduction in LARR of at least 25% may have been important in the search for preoperative predictors. This value, higher than that used in other studies, was able to overcome the intra- and interobserver variability in left atrium volume measures and may therefore be more clinically relevant.

Considering the known association between increased left atrial size and cardiac events in patients with MR [10, 11] and the fact that early surgery may avoid excess left atrial dilation, it seems reasonable to point out that LARR after MR surgery may be beneficial. A recent large multicenter study using propensity-score matching found that early surgery in patients with MR due to flail leaflets was associated with less heart failure and lower mortality than medical management. However, there was no difference in atrial fibrilation, which is usually associated with a large left atrium [28]. In our study, preoperative atrial fibrillation was not associated with postoperative LARR; of 12 patients with preoperative atrial fibrillation, 2 recovered sinus rhythm after surgery. Another retrospective study did not find association between LARR and reduction of cardiovascular events, mortality and atrial fibrillation [29]. Further studies are needed to verify the prognostic benefits of LARR after mitral-valve surgery in patients with MR.

## Study limitations

The time between postoperative echocardiography and surgery was relatively short in our study. Because of our method of patient selection, the results of this study may not be applicable to patients with MR from other causes, to patients with acute mitral insufficiency or to those with more than mild residual MR. Despite in our study the type of surgery was not related to LARR, a finding similar to other study [16], due to relative small number of patients and variations in type and size of prosthetic valves, we were not able to analyze separately patients who underwent mitral valve repair or replacement. Finally, 3-dimensional echocardiography was not used in our study, although it seems to be slightly more precise for determining LA volume compared with 2-dimensional echocardiography, and facilitates functional analysis.

## Conclusion

In patients with primary chronic severe MR, LARR occurs frequently after valvular surgery and is associated with the preoperative LVEF higher than 63.5%.
